# A Young Adult With Multisystem Inflammatory Syndrome Following Weeks of Initial COVID-19 Respiratory Infection, With No Prior COVID-19 Vaccination: A Case Report

**DOI:** 10.7759/cureus.40745

**Published:** 2023-06-21

**Authors:** Ans Ahmad, Mashal Maheshwari, Manas Gunani, Omer A Shaikh, Abdulqadir J Nashwan

**Affiliations:** 1 Medicine, Aultman Hospital, Bedford, USA; 2 Medicine, Sawai Man Singh Medical College, Jaipur, IND; 3 Internal Medicine, Ziauddin University, Karachi, PAK; 4 Nursing, Hamad Medical Corporation, Doha, QAT

**Keywords:** rt-pcr test (reverse transcription-pcr), cardiovascular risk, multisystem involvement, covid-19, multisystem inflammatory syndrome

## Abstract

Multisystem inflammatory syndrome (MIS) following COVID-19, a condition primarily diagnosed in children, has also been observed less frequently in adults. It usually presents with a multitude of symptoms, mimicking a shock-like state characterized by multiple organ failure. Diagnosis often involves ruling out other conditions and timely management to mitigate morbidity and mortality. In this case, a 39-year-old unvaccinated Caucasian male patient reported symptoms of fever, chills, night sweats, diarrhea, headache, nasal congestion, and facial pain. Despite treatment with antipyretics, the fever persisted. The patient had tested positive for COVID-19 via polymerase chain reaction (PCR) six weeks prior. Clinical findings included low oxygen saturation, sinus tachycardia, abnormal liver function, elevated inflammatory markers, a negative respiratory viral panel, a negative immunologic workup, and a positive *Clostridium difficile* (*C. difficile*) PCR. Following complaints of chest pain which quickly escalated to cardiac arrest, he was diagnosed with myopericarditis. These manifestations met the multisystem inflammatory syndrome in adults (MIS-A) diagnostic criteria as stipulated by the Centers for Disease Control and Prevention. The diagnosis of MIS-A was reached through exclusion. Notably, the patient responded well to symptomatic management. Given the infrequent occurrence of MIS-A cases, even in 2023, it remains a challenging diagnosis. Despite existing guidelines for management, the recovery of this patient solely through symptomatic treatment prior to the consideration of conventional treatment is striking. The patient had concurrent infections, including a *C. difficile* infection, but these did not account for the overall clinical presentation, particularly the myopericarditis and positive laboratory findings.

## Introduction

The ongoing COVID-19 pandemic continues to profoundly impact our lives, even amid widespread vaccination and treatment measures. The multisystem inflammatory syndrome (MIS) in children was first diagnosed in April 2020, initially misdiagnosed as Kawasaki disease or a toxic shock-like state until an accumulation of similar cases emerged [1]. This syndrome is thought to arise from an immunologic response to the severe acute respiratory syndrome coronavirus 2 (SARS-CoV-2) virus, with the cytokine storm hypothesis postulated as the underlying pathophysiology [2]. Similar cases started surfacing in adults not long after, leading the Centers for Disease Control and Prevention (CDC) to define specific criteria to guide physicians toward this unusual diagnosis [1].

Multisystem inflammatory syndrome in adults (MIS-A) often presents several weeks post-COVID-19 infection, frequently after the acute illness has resolved [3]. Symptoms may include high fever, gastrointestinal issues, rash, conjunctivitis, and severe cardiac involvement [3]. Some patients experience a severe inflammatory state, leading to multiple organ dysfunction that can resemble a shock-like state [3]. The exact pathogenesis of MIS-A is still being investigated, but it is hypothesized to be related to an overactive immune response to SARS-CoV-2, which leads to a so-called 'cytokine storm', resulting in widespread inflammation and damage to bodily tissues [2]. Diagnosing MIS-A is challenging due to its rarity and overlap with other conditions [4]. The CDC has issued guidelines, including clinical and laboratory criteria, to aid diagnosis [5]. Treatment typically involves managing symptoms and reducing inflammation, often using immunomodulatory therapies such as corticosteroids [5]. 

Our case meets both the clinical and laboratory criteria set forth by the CDC, as the patient presented with high-grade fever prior to hospitalization, cardiac involvement within the first three days of hospitalization, thrombocytopenia, shock, recent positive COVID-19 polymerase chain reaction (PCR), and elevated inflammatory markers. Although steroids remain the cornerstone of long-term care, we present here a unique case that responded to symptomatic treatment and led to the patient's subsequent discharge.

## Case presentation

A 39-year-old Caucasian male arrived at the emergency room, reporting symptoms of fever, chills, and night sweats that had begun three to four days prior. Despite self-medication with 4 g of Tylenol daily, his fever persisted. Upon examination, his fever peaked at 104.5 Fahrenheit. He also reported one to two episodes of diarrhea, facial pain in the frontal and maxillary sinus areas, headache, and nasal congestion but denied any symptoms of a sore throat, cough, chest pain, or shortness of breath. The physical examination did not reveal any abnormal findings. His medical history included a symptomatic COVID-19 infection six weeks prior, and he confirmed not receiving the COVID-19 vaccine.

The initial evaluation in the ER showed normal vital signs, aside from tachycardia with a heart rate of 139 bpm and hypoxia, marked by oxygen saturation (SpO_2_) at 89%. Blood and urine cultures were collected, and he was started on supplemental oxygen, IV fluids, and broad-spectrum antibiotics. The unremarkable chest X-ray and CT angiogram were performed to rule out pulmonary embolism due to tachycardia and hypoxia. However, a CT scan of the sinuses revealed bilateral thickening and opacification of the frontal and maxillary sinuses, warranting further hospital evaluation and management.

The patient's lab results revealed leukocytosis, thrombocytopenia, low sodium, mildly elevated liver function tests with normal bilirubin, and increased lactic acid. The respiratory viral panel and COVID-19 PCR were negative. Despite the inconclusive abdomen and pelvis CT and negative results for most immunologic and infectious disease tests, the patient tested positive for *C. difficile* PCR, leading to oral vancomycin and IV metronidazole treatment.

**Figure 1:**
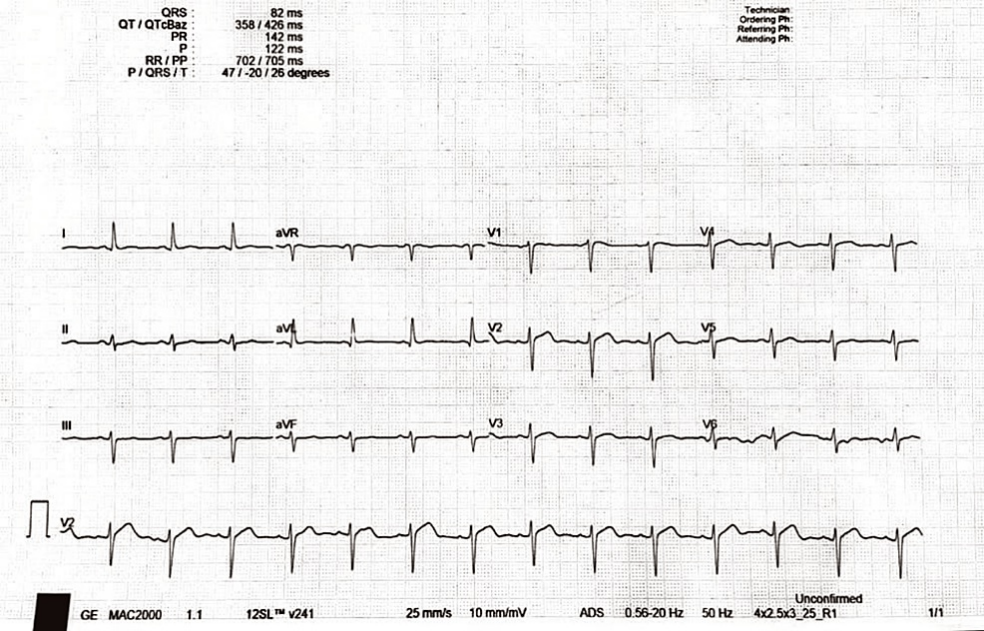
Initial ECG shows no significant abnormality other than nonspecific ST segment and T wave changes.

On the third day of hospitalization, the patient reported sharp, stabbing chest pain radiating to his back and shoulders, which rapidly escalated to cardiac arrest. The rapid response team managed the situation successfully. The ECG revealed unspecified ST segment changes; blood tests showed elevated troponin levels (Figure 1).

**Figure 2:**
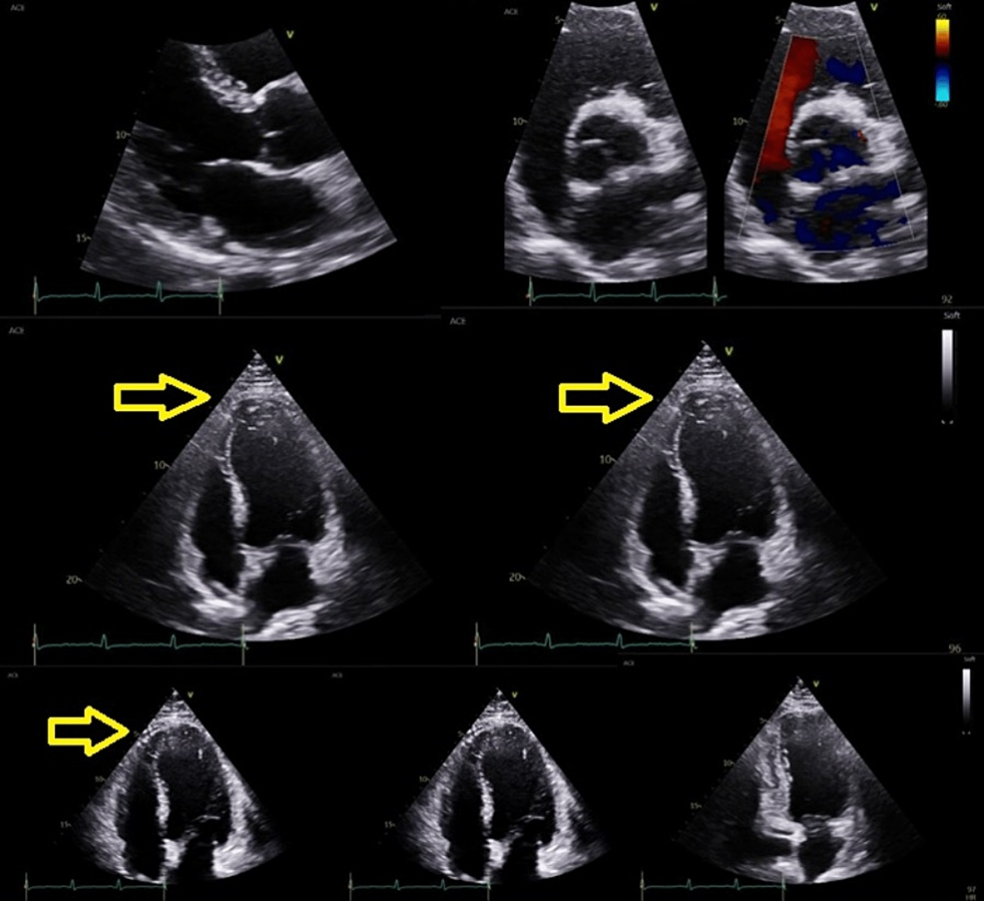
Echocardiography performed on the third day of admission shows mild nonspecific myopericardial changes (yellow arrows), normal valve thickness, and LV cavity. LV: left ventricle.

The 2D echocardiography indicated a small pericardial effusion and myocardial abnormality, with no signs of vegetation or chamber abnormalities. This suggested possible myopericarditis (Figure 2).

Due to aspirin allergies, the patient was desensitized and administered 325 mg of aspirin twice daily without adverse effects. He was also prescribed colchicine. Despite being unable to undergo a cardiac MRI due to claustrophobia, the patient's heart catheterization showed no acute blockages. He was advised to continue taking carvedilol and to schedule a follow-up cardiac MRI as an outpatient. The patient's condition improved, and he was medically stable for discharge.

## Discussion

Multisystem inflammatory syndrome in adults (MIS-A) manifests with multiple system involvement, as suggested by reported instances of patients developing acute kidney failure, cutaneous and ocular manifestations, and neurological symptoms [1,6-8]. Our case, like that reported by Chau et al. [9], experienced significant myocardial damage and cardiac arrest without any underlying cardiovascular risk factors.

The temporal relationship between the onset of MIS-A and prior COVID-19 infection remains unclear [10], although it is typically considered to occur two to 12 weeks post-infection. Thus, suspicion of MIS-A should arise in any patient with a history of COVID-19 infection presenting with multi-organ failure. Antibody titers may assist in the diagnosis, signifying the host's immunological response to the infection.

Our patient had concurrent sinusitis and a positive *C. difficile* infection, both of which were treated with broad-spectrum antibiotics. However, these findings did not entirely explain the clinical presentation, rendering MIS-A a diagnosis of exclusion after ruling out immunological, rheumatological, and infectious etiologies. Similar to the reported cases of MISC in pediatric populations, our patient also had thrombocytopenia and hepatitis, indicating parallels in the disease pathology of adults and children [11].

Effective treatment strategies for MIS-A remain uncertain. Lai et al. noted that current approaches comprise anti-inflammatory agents, including intravenous immunoglobulin (IVIG) and corticosteroids [11]. Past case reports indicate remarkable responses to IVIG [12]. Interestingly, our case demonstrated recovery with symptomatic treatment alone, suggesting that his immune response to a recent infection had naturally diminished, thus negating the need for steroid treatment. Nonetheless, had the diagnosis of MIS-A been made earlier, steroid treatment could have been initiated sooner, highlighting the importance of timely diagnosis and treatment initiation for this condition.

Furthermore, higher mortality rates have been observed in conjunction with comorbidities or cardiac involvement among patients who are hospitalized due to COVID-19 [13-14]. Notably, the patient in our case study had experienced a previous bout with COVID-19 infection but exhibited no comorbidities. This absence of additional health complications may have played a pivotal role in facilitating his recovery. Contrarily, various studies have highlighted that the onset of MIS can range from a week to as long as a month following the cessation of viral replication, and intriguingly, some of these studies have also discerned a potential association between the development of MIS and the administration of the COVID-19 vaccine [15].

## Conclusions

In conclusion, multisystem inflammatory syndrome in adults (MIS-A) is a severe post-acute complication of COVID-19. It can present with cardiac involvement and develop weeks after the initial infection. Maintaining clinical suspicion for MIS-A in COVID-19 patients with multi-organ failure is crucial. Diagnosis is challenging and requires ruling out other causes. Treatment focuses on symptom management and immunomodulatory therapies. Further research is needed to understand MIS-A's underlying mechanisms and optimize treatment strategies. Prompt management is vital for improving patient outcomes.
